# Psychometric properties of the Swedish version of the Patient Health Questionnaire-9: an investigation using Rasch analysis and confirmatory factor analysis

**DOI:** 10.1186/s12888-024-06417-4

**Published:** 2025-01-13

**Authors:** David Forsström, Farzaneh Badinlou, Magnus Johansson, Olivia Ojala, Samir El Alaoui, Kristoffer N. T. Månsson, Alexander Rozental, Johan Lundin, Simon Jangard, Shervin Shahnavaz, Karolina Sörman, Nitya Jayaram-Lindström, Tobias Lundgren, Markus Jansson-Fröjmark, Maria Hedman-Lagerlöf

**Affiliations:** 1 Department of Clinical Neuroscience, Centre for Psychiatry Research, Karolinska Institutet, & Stockholm Health Care Services, Region Stockholm, Stockholm, Sweden; 2 Medical Unit Medical Psychology, Women’s Health and Allied Health Professionals Theme, Karolinska University Hospital, Solna, Sweden; 3 Division Built Environment, System Transition, RISE Research Institutes of Sweden, Stockholm, Sweden; 4 Department of Clinical Neuroscience, Karolinska Institutet, Stockholm, Sweden; 5 Department of Health, Education and Technology, Luleå University of Technology, Luleå, Sweden; 6 Department of Clinical Neuroscience, Division of Psychology, Karolinska Institutet, Solna, Sweden

**Keywords:** Patient health Questionnaire-9, Swedish version, Psychometrics, Confirmatory factor analysis, Rasch analysis

## Abstract

Depression is one of the most common psychiatric conditions. Given its high prevalence and disease burden, accurate diagnostic procedures and valid instruments are warranted to identify those in need of treatment. The Patient Health Questionnaire-9 (PHQ-9) is one of the most widely used self-report measures of depression, and its validity and reliability has been evaluated in several languages. However, the Swedish translation has yet not been subject to psychometric evaluation, and no previous psychometric evaluation of the instrument have used both Rasch analysis and classic test theory. The aim of this study was to investigate validity and reliability of the PHQ-9 in a Swedish sample of individuals with self-reported current or past mental health problems using Rasch analysis and confirmatory factor analysis. A sample of 4958 participants was recruited from an online survey covering different aspects of covid-19 and mental health targeted towards individuals with mental health problems. Results showed that a one-factor solution fit the data after removing one item and that some items had a different response pattern for older respondents. Furthermore, after removing item 2 and merging the two middle response categories for item 9, the Swedish translation of PHQ-9 showed adequate psychometric properties. The findings suggests that the Swedish translation of PHQ-9 may need to be adapted in order to make adequate interpretations of an individual score and to differentiate between populations. Further tests of its validity and reliability in other Swedish samples are needed to fully understand the properties of the Swedish version of PHQ-9.

**Supplementary Information:**

The online version contains supplementary material available at 10.1186/s12888-024-06417-4.

## Introduction

Depression is one of the most prevalent psychiatric conditions, affecting people across the globe and the lifespan. It is a leading cause of disability worldwide, and a major contributor to the overall global burden of disease [46]. Mood disorder is the umbrella term for the different sub-categories of depression in The Diagnostic and Statistical Manual of Mental Disorders, Fifth Edition (DSM-5); [6], of which major depressive disorder (MDD) is the most common [20, 62]. While most studies have investigated the level of depression or depressive symptoms in specific sub-populations, e.g., diabetes [24], dementia [43], or cancer [55, 64], some studies have investigated depression in the general population. A meta-analysis evaluating the aggregate prevalence of depression within the general population between 1994 and 2014 showed a point- and lifetime prevalence of 12.9 and 10.8% respectively, with a higher point prevalence in women (14.4%), in countries with a medium human development index (29.2%) and studies using self-report instruments (17.3%) to assess depression [48]. In a Swedish context, the prevalence of clinically significant depression was found to be 10.8%, and the point prevalence of MDD was 5.2% [38]. However, using self-report instruments can induce response bias, with response rates contributing to heterogeneity. In summary, the available literature indicating a high prevalence of depression, and given that prevalence rates may differ as a function of measurement methods, points to a need for instruments that can adequately assess the level of depression in different populations. One widely used instrument is the Patient Health Questionnaire 9 (PHQ-9), originally developed and psychometrically tested by Kroenke et al. [41]. The instrument has been psychometrically evaluated in many languages and in different populations [23, 27, 49, 54, 60, 75], and has shown satisfactory psychometric properties and adequate validity as a measure of depression [28, 41]. Moreover, efforts have been made to evaluate the cross-language item functioning of the PHQ-9 by comparing populations that speak different languages [7]. The instrument has been tested in primary care as well as psychiatric outpatient populations [9, 59, 65, 75], in other modalities, such as via computer or automated telephone calls [23, 25], and also in resource-constrained settings [13]. The Swedish version has been around since 2010, was translated by mats Adler, and evaluated regarding its clinical use (Adler et al.) and psychometric properties [1, 30]. However, no study has thoroughly evaluated PHQ-9 in a large sample and using both Rasch-analysis and confirmatory factor analysis.

Historically, classical test theory has been the predominant approach for psychometric evaluation [4, 61]. However, alternative analytic strategies such as Rasch analysis [11, 66] enables a more elaborated investigation of measurement functioning (e.g., investigating strengths and weaknesses of an instrument, such as evaluating how well test items are defining a given variable). For instance, Lambert et al. [44] compared several widely used depression questionnaires including the PHQ-9 using Rasch analysis, and found that the labels of mild, moderate, and severe depression attributed to the cut-off scores across these scales were not equivalent. That is, at least some of the variability in prevalence estimates of depression appears to be caused by measurement artefact, a finding that needs to be taken into account when interpreting prevalence estimates across studies.

A psychometric evaluation of the Swedish version of PHQ-9 is relevant for several reasons. Results from earlier studies are mixed; some studies have demonstrated a one-factor structure [34, 35, 45], whereas others have found a multidimensional structure [26, 33, 40]. The instrument is widely used in research studies in Sweden, and one of the most commonly administered instruments within health care settings such as primary care and outpatient psychiatry as well as in research studies. In addition, due to the widespread use of the instrument, exploring the instrument using a combination of classic test theory with Rasch analysis could provide a more detailed understanding of the instrument in a psychiatric population.

The aim of the current study is to examine the validity and reliability of PHQ-9 in a sample of individuals living in Sweden, with self-reported current or past mental health problems. Understanding how the instrument functions for individuals with mental illness is an important step towards understanding the applicability of the scale in Swedish context. Without a comprehensive psychometric evaluation there is a risk the scores can be misinterpreted. The analytic procedure includes Rasch analysis and confirmatory factor analysis to examine psychometric properties of the instrument. These methods are commonly used in field of psychometrics, which enables comparison of the results with previous studies.

## Materials and methods

### Participants

The current study is part of a larger longitudinal study (*N* = 6095) [68] evaluating the impact of COVID-19 on individuals with mental health problems. For the purpose of the present research question, 4958 individuals contributing to complete baseline data were included. Inclusion criteria were: (1) being 18 years or older, (2) living in Sweden, (3) having a current or lifetime diagnosis of at least one psychiatric disorder. About half of the participants had a university education, and the majority were working full or part-time and were born in Sweden (see Table 1). The study was approved by the Swedish Ethical Review Authority (Dnr 2020–02798 and 2021 − 00521) and all participants provided informed consent. The legislation regulating the Swedish Ethical Review Authority and the approval from the Swedish Ethical Review Authority is in compliance with the Helsinki Declaration.

**Table 1:** Demographics of the sample (*N* = 4958)

Age, M (SD)	35.7 (12.3)
Sex, *n* (%)	
Male	1168 (23.4)
Female	3568 (71.6)
Other	249 (5.0)
Education (highest level), *n* (*%*)	
Elementary school	351 (7.0)
High school	1968 (39.5)
University	2666 (53.5)
Work status, *n* (*%*)^a^	
Working full-time or Part-time	3403 (68.3)
Unemployed	546 (11.0)
Retired	384 (7.7)
Sick leave	197 (4.0)
Parental leave	96 (1.9)
Student	1217 (24.4)
Other	344 (6.9)
Birthplace, Sweden, *n* (*%*)	4654 (93.4)

^a^Allowed for multiple response options

Clinical characteristics of the study population are presented in Table 2. In the total sample, 93.5% of participants reported at least one life-time mental health problem from a list of common psychiatric disorders, with the average number of psychiatric diagnoses per individual being 3.13 (*SD* = 2.06). A majority of the sample 83.8% (*n* = 4176) reported being diagnosed with major depressive disorder. Moreover, 45.7% reported that they had been diagnosed with generalized anxiety disorder and 42.0% with panic disorder.

**Table 2:** Self-reported lifetime mental health problems

	*n*	%
Bipolar and Related Disorders	925	18.6
Major Depressive Disorder	4176	83.8
Anxiety Disorders		
Specific Phobia	238	4.8
Social Anxiety Disorder	948	19.0
Panic Disorder	2095	42.0
Agoraphobia	214	4.3
Generalized Anxiety Disorder	2278	45.7
Somatic Symptom and Related Disorder	332	6.7
Obsessive-Compulsive and Related Disorders	454	9.1
Trauma- and Stressor-Related Disorders	1040	20.9
Feeding and Eating Disorder	1056	21.2
Schizophrenia Spectrum and Other Psychotic Disorders	217	4.3
Substance-Related and Addictive Disorders	472	9.5
Neurodevelopmental Disorders	1199	24.1
None of above	293	5.9
I don’t know	84	1.7

Allowed for multiple response options

### Procedure

This study is based on survey data collected in Sweden between July 2020 and July 2021. Participants [68] were recruited via a study website, through social media and through collaborations with psychiatric clinics who assisted in spreading information about the study. Eligibility criteria were as follows: (1) being 18 years or older, (2) living in Sweden, and (3) having a current or lifetime experience of psychiatric symptoms. The participants reported the problems they faced by ticking of a list with common mental disorders. Study data was collected and managed via Research Electronic Data Capture (REDCap), an electronic data collection service, hosted at the Karolinska Institutet [31,32].

## Measures

### Demographic variables

Demographic information about the participants in the current study consisted of age, sex, education, work status, and place of birth.

### Lifetime mental health problems

Lifetime mental health problems was measured with checklist, in which respondents stated whether they had ever been diagnosed with a mental health problem. Respondents were allowed to choose more than one alternative (see Table 2).

### Depression

The PHQ-9 is a self-report instrument which measures depressive symptoms [41]. The instrument consists of nine items corresponding to major depressive disorder criterion according to the Diagnostic and Statistical Manual-IV [5] and respondents are instructed to indicate how often they have been bothered by nine different symptoms during the past two weeks, on a 4-point Likert scale from 0 (“*not at all*”) to 3 (“*nearly every day*”). Possible ordinal sum scores on the PHQ-9 range from 0 to 27. The Swedish translation was carried out by a professor in psychiatry, but no testing was carried out when the instrument was translated to Swedish. Of note, one item got a slightly different wording in the Swedish translation. The Swedish translation reads “*Feeling down*,* depressed or felt that the future looks hopeless?*”, thus adding a sub-phrase regarding the future.

## Analysis of data

Descriptive statistics were performed using SPSS 28.0 to display the demographic and clinical characteristics of the sample. There were no missing data since only respondents answering all questions of PHQ-9 were used.

The Rasch analysis was structured to assess four criteria necessary to justify the sum score as a sufficient metric of the latent variable: unidimensionality; local independence; ordered response categories (monotonicity); and invariance [16]. Also, reliability, targeting, and person fit was assessed and reported.

The Rasch model makes it possible to estimate both item probabilitythresholds and respondents locations on latent continuum Additionally, properties of the items compared to the respondents was reported by targeting and reliability figures and descriptives.

Dimensionality was examined by using several methods. A Principal Component Analysis (PCA) examining the Rasch model residuals was carried out. Eigenvalues below 2.0 support unidimensionality [12]. Item fit was checked by using weighted and unweighted mean square statistics (MSQ) and z-standardized statistics (ZSTD). These values should be within the range of 0.7–1.3 and +/- 1.96, respectively [10]. In large samples it is common for ZSTD to be inflated [29], resulting in the occurrence of type-1-errors in large samples. Thus, 50 randomly chosen subsamples of *n* = 250 were analyzed and their average ZSTD values were calculated.

Local independence was assessed by evaluating Yen’s Q3 residual correlations between item pairs. The *r* value should not exceed 0.2 above the mean of all item-pair residual correlations [17].

The distribution of response scale steps for the individual items was analyzed by visualizing response category probability distributions. Item category thresholds are the locations on the latent continuum where two adjacent response category probability curves cross. Visual inspection of item characteristic curves was performed to understand the probability of different scale steps and to check whether item threshold locations were disordered. Each item response category should at some point of the latent continuum be the most probable response in order to provide meaningful information.

Invariance was investigated by assessing potential differential item functioning (DIF) related to age and sex, with 0.5 logits as a DIF magnitude cutoff value [39]. The sample of *n* = 4985 is large enough to be able to perform a well-powered DIF analysis for sex and age.

Mean and standard deviations were calculated for both item and person location distributions. Person locations are the estimated “factor scores” on the interval level scale of the latent continuum. In the Rasch model, properties of items and persons are estimated on the same scale, which is illlustrated by a targeting figure that shows persons’ and item thresholds’ locations in parallel. Reliability was examined by using test information function (TIF) curve by reporting the proportions of respondents located within the scale range where reliability was estimated at or above person separation index 0.7. Point estimate of reliability was not reported as it is more relevant to report the TIF, since the TIF reports item properties rather than sample properties [39]. Person fit was assessed using weighted (infit) ZSTD, which similarly to items should be in the range of +/- 1.96.

The Rasch partial credit model with conditional maximum likelihood estimation was applied, using the R package eRm version 1.0–2 [51, 52]. DIF analysis was carried out by using the R package psychotree version 0.16-0 [72]. The R package mirt version 1.37.1 [14] was used to analyze response categories and residual correlations. Figures and tables were made by applying the R package RISEkbmRasch version 0.1.8.4 [36, 37]. An analysis report was created using Quarto [3], containing analysis code which can be found on the Open Science Foundation website by following the URL provided under the Additional Materials section. The analyses were carried out by using R version 4.2.2 [18] and Rstudio version 2022.12.0 [2]. Confirmatory factor analysis was carried out using the lavaan software package for R, version 0.6–12 [67] with the Weighted Least Squares Mean and Variance adjusted (WLSMV) estimator [47].

## Results

### Rating scale

**Fig. 1:**
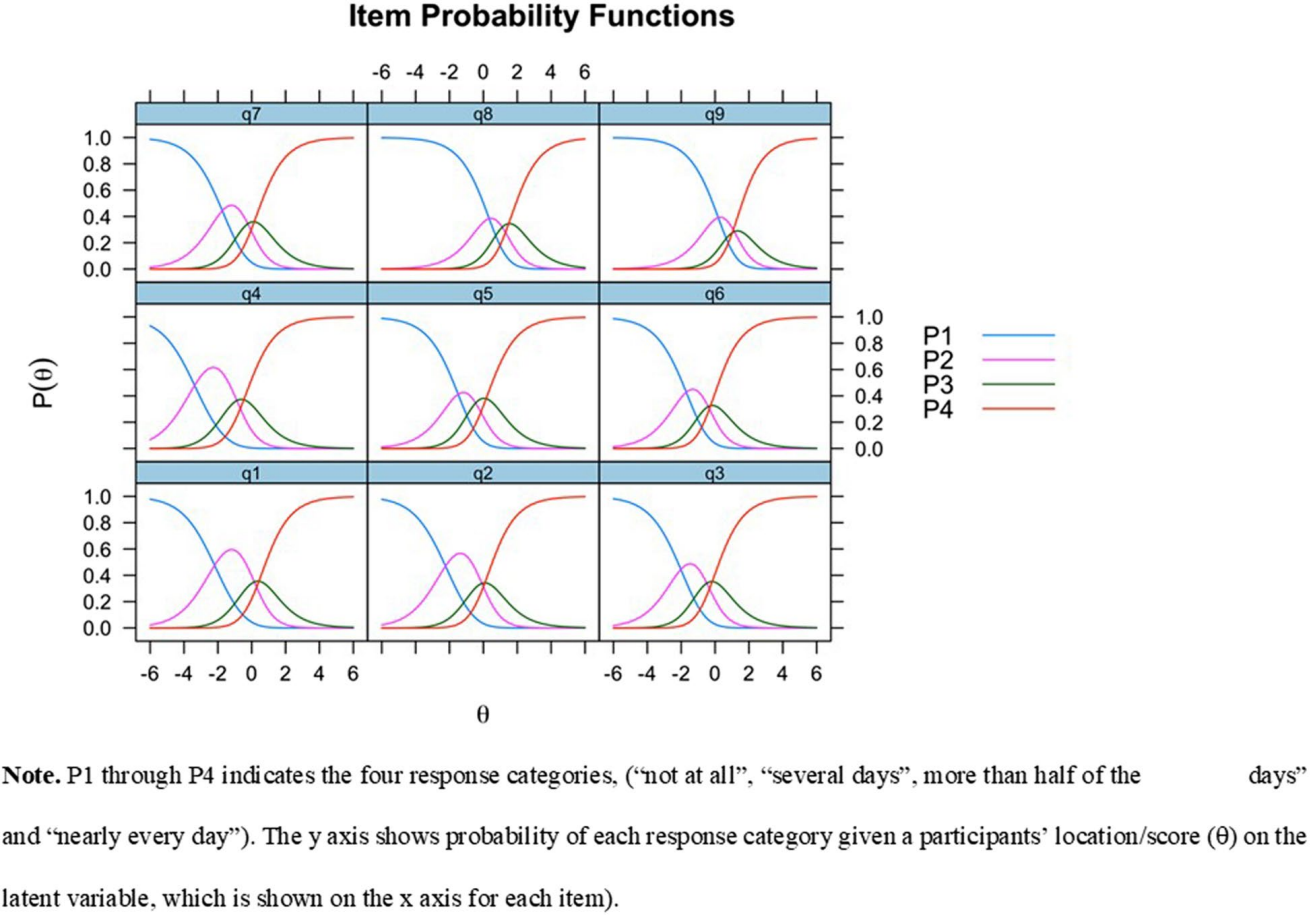
Item probability functions

Figure 1 shows the response category probabilities. While several items had very small distances between the higher item category thresholds, item 9 was the only one that showed disordered response categories (endorsed in the wrong order). The probability curve for item 9’s second highest response category indicated that it was not the most likely response category at any point of the x axis (θ). After merging the two middle response categories for item 9 (*several days* and *more than half of the days*, respectively) the response categories were no longer disordered.

### Distribution of response categories

**Fig. 2:**
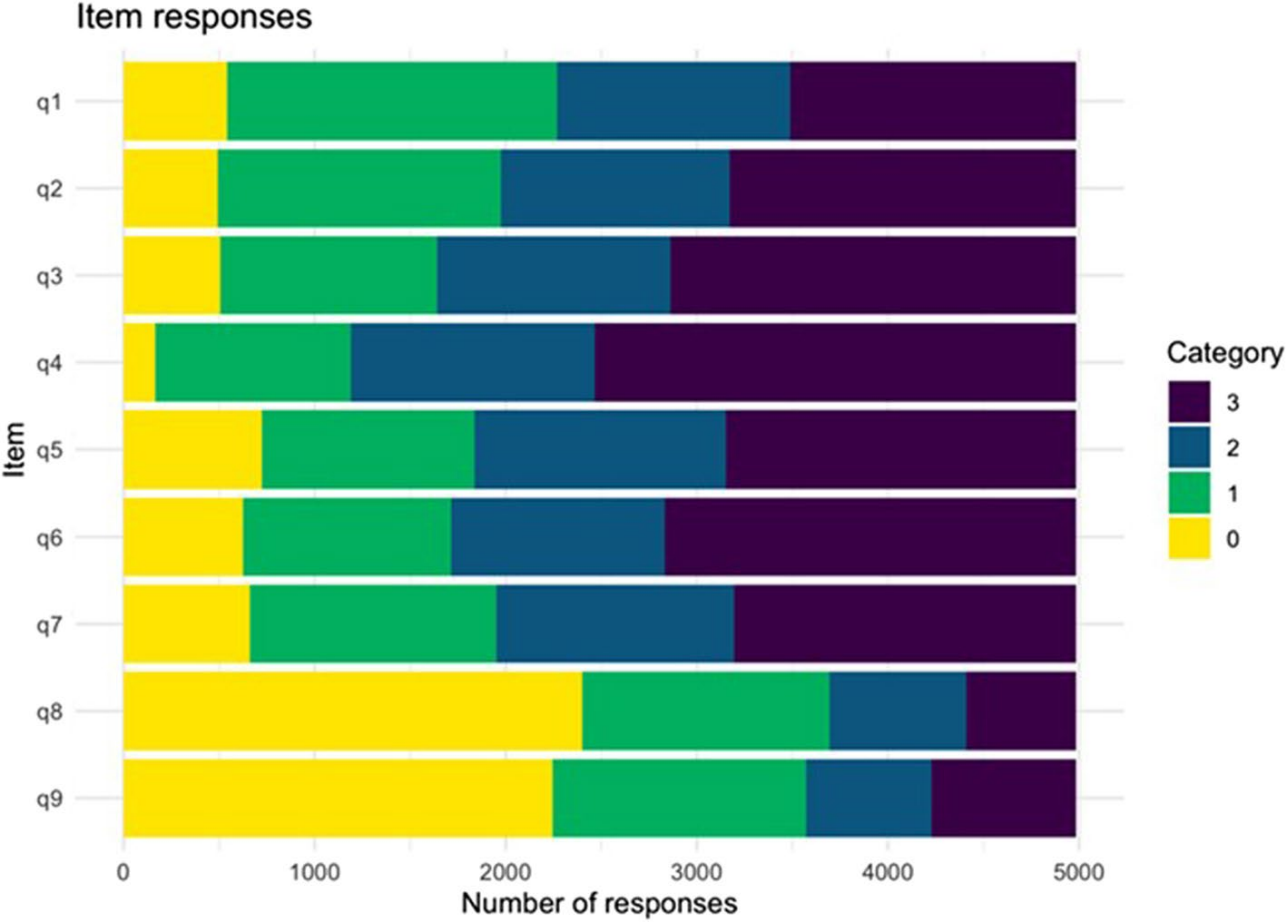
Distribution of response categories

The total number of responses to each response category for all items indicated that item 8 and item 9 were less endorsed than the remaining seven items. This indicates that the items 8 and 9 might indicate more severity (Fig. 2).

### Scale dimensionality

**Fig. 3:**
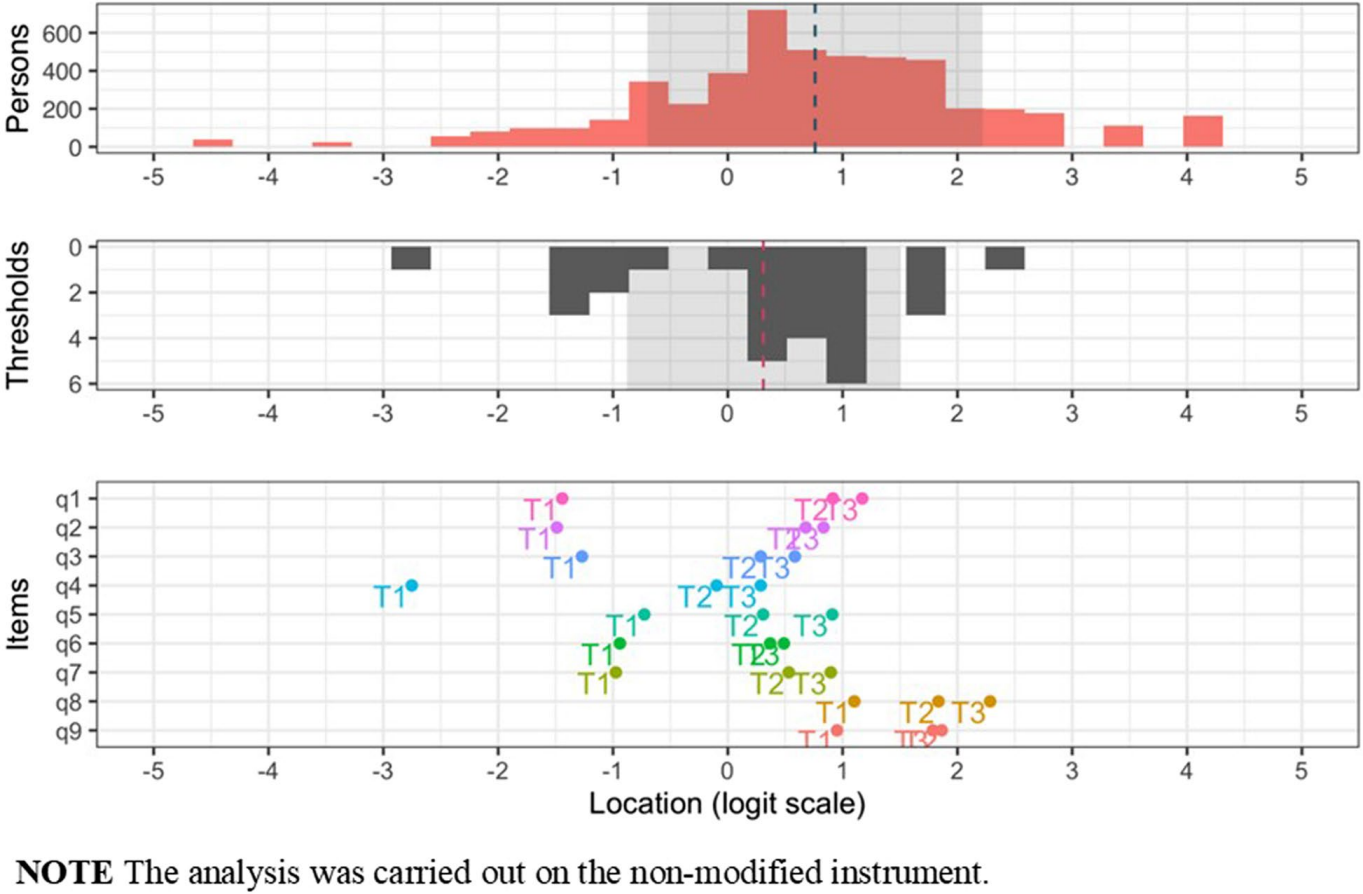
Item threshold and person locations (targeting)

The largest Eigenvalue from the PCA analysis of standardized Rasch residuals was 1.74. Pair-wise residual correlations showed an average level of −0.11, with two item-pairs having correlations more than 0.20 above the average (cutoff = 0.09). Items 1 (“*Little interest or pleasure in doing things*”) and 2 (“*Feeling down*,* depressed*,* or hopeless*”) correlated at 0.27, while items 3 (“*Trouble falling or staying asleep or sleeping too much*”) and 4 (“*Feeling tired or having little energy*”) correlated at 0.11. The residual correlation between items 1 and 2 was sufficiently large to cause issues with unidimensionality. Combining the two items into a “super-item” could be a feasible solution but would be inappropriate due to the similar item threshold locations (see Fig. 3). This has been done in previous studies with an analytic stance based on the Rasch model [50, 56, 57]. One of the items were removed to fulfill the criteria for unidimensionality. Since item 2 showed relatively low item fit, it was removed from the item set and item 1 retained. Running the Rasch analysis again without item 2 showed that the residual correlation between items 3 and 4 was no longer above the cutoff, while items 6 and 9 were now correlated 0.018 above the cutoff, which was deemed acceptable. See Supplementary Materials 1 for a table illustrating the key results in the two iterations of the Rasch analyses.

Item fit metrics are shown in Table 3. Item 2 and 4 show low levels of item fit for both infit MSQ and infit ZSTD.

**Table 3:** Item fit values

Items	OutfitMSQ	InfitMSQ	OutfitZSTD	InfitZSTD
q1	0.975	0.937	−0.254	−0.426
q2	0.616	**0.657**	−4.227	**−4.599**
q3	1.085	1.05	0.814	0.477
q4	0.664	**0.733**	−3.11	**−3.149**
q5	0.964	0.962	−0.181	−0.409
q6	0.822	0.861	−1.723	−1.675
q7	0.94	0.941	−0.592	−0.704
q8	1.153	1.132	1.115	1.134
q9	1.009	1.068	0.002	0.557

*Abbreviations:* *MSQ* Mean square statistics, *ZSTD* Z-standardized statistics

### DIF analysis

Analysis of differential item functioning (DIF) for sex indicated that items function similarly for men and women, with no item location differences larger than 0.3 logits. DIF for education level also showed unproblematic differences. For age-based DIF analysis, the recursive tree method used showed that item 6 (“*Feeling bad about yourself – or that you are a failure or have let yourself or your family down*”) had a 0.58 logits higher item location for ages above 59 (*n* = 245) compared to younger participants (age ≤ 42). The largest item location differences between age groups were found for items 1 and 8 were above 0.40 logits. These also involved the age group > 59, especially compared to those younger than 42 and 28, respectively.

Analysis of person fit showed that 90.2% of participants had an infit ZSTD value between +/- 2.0.

### Reliability

**Fig. 4:**
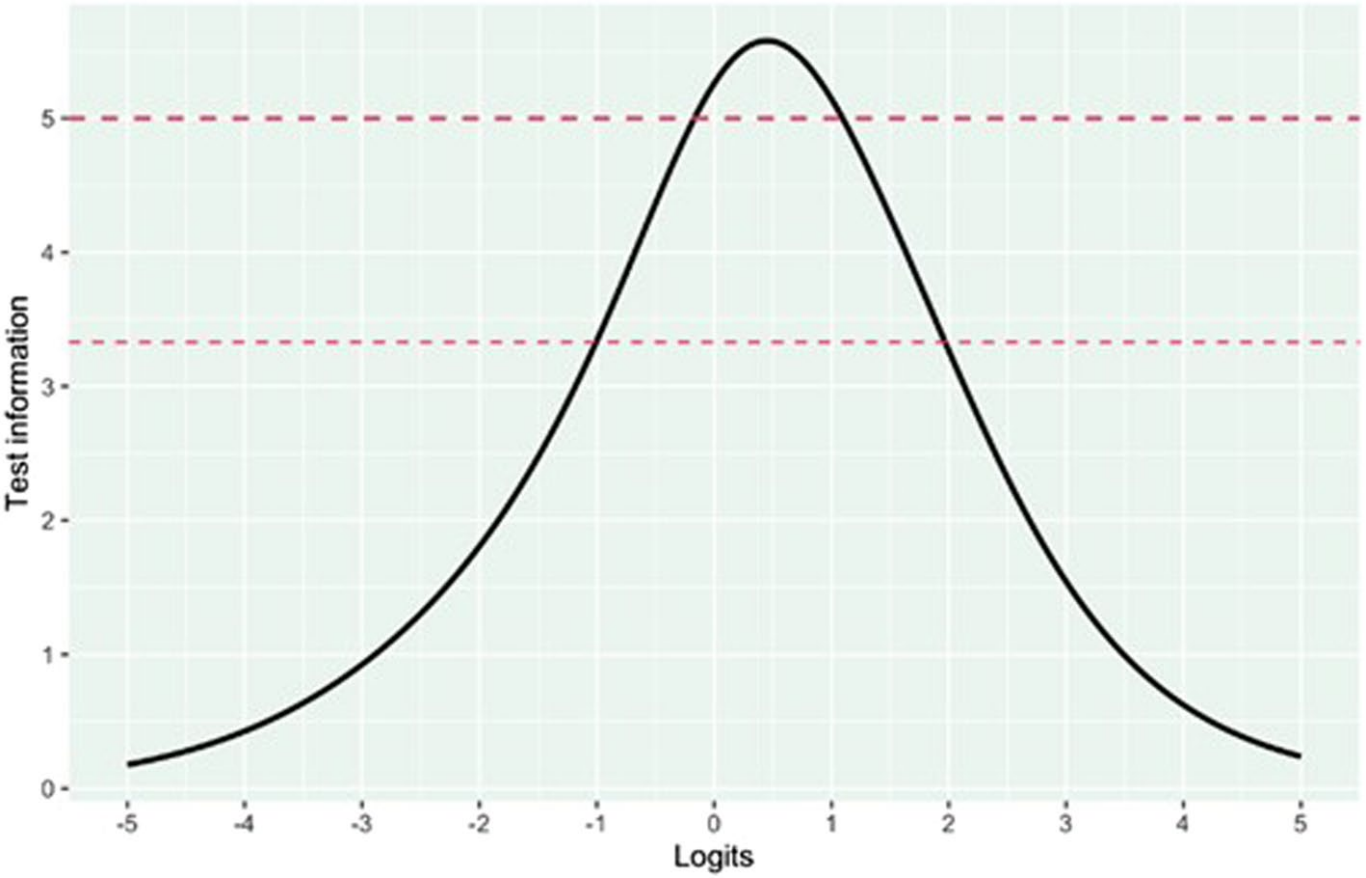
Reliability shown by a test information curve

The test information curve (see Fig. 4) showed that reliability was highest in the middle of the scale. The Person Separation Index (PSI), indicating how efficiently a set of items was able to separate individuals measured on a continuum, of 0.7 was reached between − 0.1 and 1.95 logits, where 72.5% of participants were located. 20.4% of participants were located above the upper threshold (1.95 logits) and 7.1% below the lower threshold (−0.1 logits) (Table 4).

**Table 4:** Item location parameters

Item	Threshold 1	Threshold 2	Threshold 3	Item average location
q1	−1.36	0.93	1.18	0.25
q3	−1.18	0.32	0.59	−0.09
q4	−2.62	−0.05	0.30	−0.79
q5	−0.65	0.33	0.92	0.2
q6	−0.86	0.40	0.50	0.01
q7	−0.90	0.56	0.90	0.19
q8	1.12	1.84	2.32	1.76
q9	0.71	2.58		1.64

Item 9 has only two thresholds

### Item difficulty

**Fig. 5:**
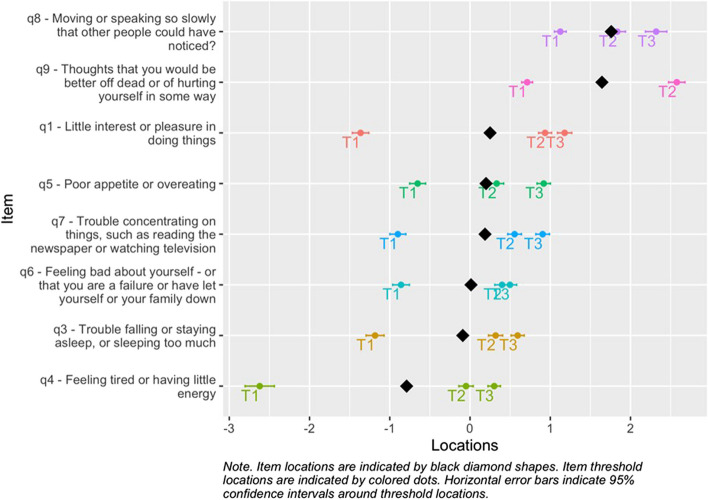
Item location

The results indicate that endorsing items 8 or 9 indicate high severity while endorsing item 3 and 4 is rather common. Item endorsement encompasses feeling tired and /or sleeping too little. The rest of the items reflect a lack of concentration indicating a lack of energy and enjoyment. This culminates in item 9 where thoughts about being dead is present. Item 9is indicative of severe depression (see Fig. 5). A table with all item threshold locations is available as Supplementary Material 2.

### Confirmatory factor analysis

**Fig. 6:**
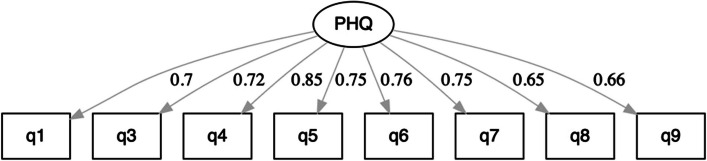
Standardized factor loadings

The confirmatory factor analysis showed inadequate fit in relation to fit indices benchmarks for the unmodified Swedish version (see Table 5), but with clear improvements for all fit metrics when item 2 has been removed. The residual correlation between items 6 and 9 was also shown in the modification indices with a *X* ^2^ of 139. For the final model, with item 2 removed and item response categories merged according to the Rasch analysis, model fit was further improved. Standardized factor loadings range between 0.65 and 0.85 (see Fig. 6).

**Table 5:** Model fit indices

Model	Chi2	df	*p*	CFI^1^	TLI^2^	RMSEA^3^	SRMR^4^
All items	2067.211	27	0	0.959	0.945	0.123	0.055
Item q2 removed	903.105	20	0	0.973	0.962	0.094	0.044
Ordered thresholds for item q9	858.856	20	0	0.974	0.963	0.092	0.043

^1^ Comparative fit index

^2^ Tucker–Lewis index

^3^ Root mean square error of approximation

^4^ Standardized root mean square residual

## Discussion

The aim of the study was to psychometrically evaluate and provide a comprehensive psychometric evaluation of the Swedish translation of PHQ-9 using confirmatory factor analysis and Rasch analysis. The main findings were that a one-factor solution did not achieve adequate model fit. However, removing item 2 and merging the two middle response categories for item 9 resulted in satisfactory model fit for both Rasch and CFA. Furthermore, there was a general lack of differentiation between response categories for almost all items, where the second highest category barely provides any additional information about respondents. These findings show that the Swedish translation of the instrument could benefit from being revised in order to achieve improved psychometric properties. First, the number of items may need to be reduced with item 2, or alternatively, evaluating a revised version of the item more closely aligned to the original wording. However, changing the wording back to the original meaning might not necessarily lead to an adequate model fit. Second, the improved fit of data when merging the response categories for item 9 is suggestive that a rewriting of the response categories should be considered, as a merge only would be difficult to read (“*several days*” and “*more than half of the days*”, respectively). A suggestion would be to add the response category “*almost every day of the week*”.

DIF analysis showed that item 6 functions differently for individuals over 59 years of age compared to those of age 42 or lower. The DIF magnitude value slightly above the cutoff (logit value = 0.5), which indicates that the DIF is unlikely to have a significant impact on the comparability of estimated person scores on the latent variable continuum.

When examining the response categories, there were small distances between item threshold locations for almost all items when it comes to response category two (“*more than half of the days*”) and three (“*nearly every day*”). A revised or reworked version of the response categories could improve differentiation between them. A newly formulated response scale with more categories might be a step forward to achieve a higher degree of differentiation and reliability and might also improve the functioning of the scale for different populations; e.g., individuals that display symptoms at a subclinical level where more differentiated information might give a more comprehensive understanding of the symptoms. Several studies have found that increasing the number of scale steps can improve scale functioning and validity [8, 53, 73]. One study found that instruments with fewer scale steps led to more extreme values [21] indicating that few scale steps can impact accuracy. Redefining the scale steps could be a way to create a new instrument based on the current version of PHQ-9. This might improve the scale functioning for a novel scale.

Also, several items are double-barreled, which means that the respondent can endorse one out of two sometimes qualitatively different statements in one item (for example item 3: “*trouble falling or staying asleep or sleeping too much*”; or item 5: “*poor appetite or overeating*”). The use of double-barreled items can result in an underestimation of symptoms, since both symptoms could be endorsed depending on symptomology of the individual responding to the questions, and whether the symptoms have fluctuated over time. Hence, this could lead to and underestimation of symptoms and thus result in a lack of accuracy.

Concerning the factor structure of the instrument, the primary factor analysis with nine items did not support a unidimensional factor structure due to the strong residual correlation between items 1 and 2. As mentioned above, previous results are mixed with some studies endorsing a one factor solution [34, 35, 45], and others a multidimensional structure [26, 33, 40]. Item 2 had low item fit values (i.e. “overfitting” the Rasch model), which is not unexpected for an item broadly targeting the latent construct (“*Feeling down*,* depressed or felt that the future looks hopeless?*”). Earlier studies have also found item 2 to have low item fit in the English [70] and Danish [15] version of PHQ-9, and similar findings have been reported for the Major Depression Inventory’s item “*Have you felt low in spirits or sad?*” in the Danish version [15, 63]. After removing item 2, the fit indices for the one factor structure improved and unidimensionality was shown tenable in both analytical methods applied. It should also be noted that low item fit is a good indicator for single-item selection [22] and items 1 and 2 are indeed used as the short screening instrument PHQ-2, which has been psychometrically tested in several languages and for different populations [19, 42, 58, 69, 71, 74].

In summary, the consequence of the present response categories is that it impacts the validity of the Swedish version of the instrument in terms of model fit and practical functioning of the scale. More specifically, these factors make it difficult to differentiate between populations and possibly hinders adequate interpretations of an individual score.

### Practical implications

The results of our analysis show that the Swedish version of the PHQ-9 should be slightly modified to fulfill basic psychometric criteria. In practice, this means removing item 2 and merging the two middle response categories for item 9 when scoring the PHQ. Ordinal sum scores should be transformed to interval scores according to the lookup table in Supplementary Materials 3 prior to use in statistical analysis. While reliability is adequate for a majority of respondents in the current sample, there is more uncertainty in the measurement of respondents with higher levels of depression (above 2 logits on the interval scale continuum).

### Limitations

The psychometric evaluation was carried out in a population that was surveyed during the COVID-19 pandemic. This fact might have resulted in skewed data with elevated rates of depression. However, since the aim of the current study was to test the psychometric properties of the PHQ-9, this might not have had a major negative influence on the results as the participants are recruited from the same time period, hence the results are based on those participants. The majority of the analytic procedures is based on the consistency of the answers, and as long the participants answered the questions in a consistent manner according to their symptom level, the fact that the data collection was carried out during the COVID-19 pandemic should not constitute a major problem. Nevertheless, investigating the Swedish version of PHQ-9 in other samples will contribute to a more detailed understanding of the psychometric properties of the instrument in a Swedish context.

Furthermore, there is a possibility that the participants did not adhere to inclusion criteria that they needed to have at least one psychiatric disorder to be eligible to participate in the study. Using clinical ratings alongside the self-reported ratings would have increased the reliability of the results. Lastly, self-report bias is always a risk when potential respondents can choose to answer a survey and report their own symptom level. This limitation is present in our study as well.

### Future research

Future research on the PHQ-9 in a Swedish setting should primarily focus on developing a new set of response categories and exploring items that target higher levels of depression to remedy the ceiling effects found. Splitting of double-barreled items could also be considered to better understand the impact of these items and investigate the potentially added information or finding justification for keeping the items as they are. Item 2 could be revised and evaluated using a version of the item more closely aligned to the original wording. This would ideally be done in combination with cognitive interviews or a similar qualitative method to bring the respondents into the development process. Another important endeavor could be to investigate potential biases in different populations (e.g., whether individuals with and without depression and mental illness understand and interpret the questions included in the instrument differently). Measurement invariance should be explored to understand if different populations respond differently to the questions. There is also a need to explore the test-retest properties of the instrument to analyze invariance over time. This would inform the quality of PHQ-9 when it comes to sensitivity to change when examining individual change scores in both clinical and research settings. Also, data from a new sample where the original wording of item 2 is used might improve the model fit of the instrument. It would be beneficial to do a new translation of the scale from English to Swedish and then back to English before collecting new data, to make sure the items cover the intended construct of the English version of the scale. The instrument can also be investigated using a qualitative methodology using the think aloud technique to understand how Swedish native speakers understand the questions included. That might, in turn, increase the possibility to adapt the instrument to a Swedish context alongside new psychometric studies.”

## Conclusions

This study shows that the psychometric properties of PHQ-9 were not satisfactory when using a Swedish psychiatric sample. The Swedish version of the instrument need adaptations to achieve adequate psychometric properties to be used in a sample similar to the one investigated in the present study. Adjusting the number of items and increasing the number of response categories would improve the scale on an overall level. The findings of the current study call for replication in other samples in a Swedish context. From a practical perspective, results from the instrument should be interpreted with caution.

## Supplementary Information

Supplementary Material 1.

## Data Availability

Data will be made avaialable if requested from the first author.
