# Supplementary material for: Psychometric properties of the Swedish version of the Patient Health Questionnaire-9: an investigation using Rasch analysis and confirmatory factor analysis
Source: BMC Psychiatry. 2025 Jan 13;25:36. doi: 10.1186/s12888-024-06417-4 (PMC11727168; doi:10.1186/s12888-024-06417-4)
Supplement: Supplementary file 1 — Supplementary Material 1. [file 12888_2024_6417_MOESM1_ESM.docx]

**Supplementary Material 1**. Iterations of Rasch Analyses Summary (table adapted from RULER guidelines)

| **Analysis** | **Items** | **Rating scale categories** | **Person mean (SD) logits** | **Item mean (SD) logits** | **Floor effect**  **%** | **Ceiling effect**  **%** | **Residual correlation pairs over 0.2 (n)** | **PSI*** | **Items with disordered thresholds**  **(n)** | **Misfitting items**  **(n)** | **PCAR**  **Eigenvalue**  **1^st^ contrast**  **(%)** | **Misfitting persons (infit ZSTD)**  **(%)** |
| --- | --- | --- | --- | --- | --- | --- | --- | --- | --- | --- | --- | --- |
| PHQ-9, all items (n = 4985), Partial Credit Model | 9 | 36 | 0.76 (1.46) | 0.31 (1.19) | 0.8% | 3.25% | 2 | 0.85 | 1 | 0 | 1.74 (19.1%) | 11.8% |
| Item 2 removed, item 9 middle response categories merged | 8 | 31 | 0.81 (1.45) | 0.4 (1.2) | 0.8% | 3.27% | 1 (barely) | 0.82 | 0 | 0 | 1.52 (19.1%) | 10.1% |

Abbreviations: PCAR=Principal Components Analysis of Residuals, PSI=Person Separation Index

*PSI calculated according to Wright & Stone (1999)

|  | Threshold 1 | Threshold 2 | Threshold 3 |
| --- | --- | --- | --- |
| PHQ item 1 | -1.37 | 1.00 | 1.31 |
| PHQ item 2 | -1.42 | 0.76 | 0.96 |
| PHQ item 3 | -1.20 | 0.36 | 0.70 |
| PHQ item 4 | -2.68 | -0.03 | 0.39 |
| PHQ item 5 | -0.66 | 0.38 | 1.03 |
| PHQ item 6 | -0.87 | 0.44 | 0.60 |
| PHQ item 7 | -0.90 | 0.61 | 1.02 |
| PHQ item 8 | 1.18 | 1.96 | 2.50 |
| PHQ item 9 | 0.77 | 2.73 | NA |

**Supplementary Material 2**. Item category threshold locations

| **Ordinal sum score** | **Interval (logit) score** | **Logit std.error** |
| --- | --- | --- |
| 0 | -4.000 | 1.530 |
| 1 | -2.776 | 0.962 |
| 2 | -2.071 | 0.761 |
| 3 | -1.582 | 0.652 |
| 4 | -1.208 | 0.582 |
| 5 | -0.904 | 0.532 |
| 6 | -0.647 | 0.496 |
| 7 | -0.422 | 0.470 |
| 8 | -0.219 | 0.451 |
| 9 | -0.030 | 0.438 |
| 10 | 0.148 | 0.429 |
| 11 | 0.321 | 0.424 |
| 12 | 0.491 | 0.424 |
| 13 | 0.663 | 0.426 |
| 14 | 0.839 | 0.432 |
| 15 | 1.022 | 0.442 |
| 16 | 1.216 | 0.456 |
| 17 | 1.425 | 0.476 |
| 18 | 1.655 | 0.502 |
| 19 | 1.915 | 0.539 |
| 20 | 2.219 | 0.594 |
| 21 | 2.602 | 0.683 |
| 22 | 3.155 | 0.861 |
| 23 | 4.306 | 1.466 |

**Supplementary Material 3**. Transformation table for ordinal sum score to interval score

**Supplementary Material 4**. Item hierarchy

**
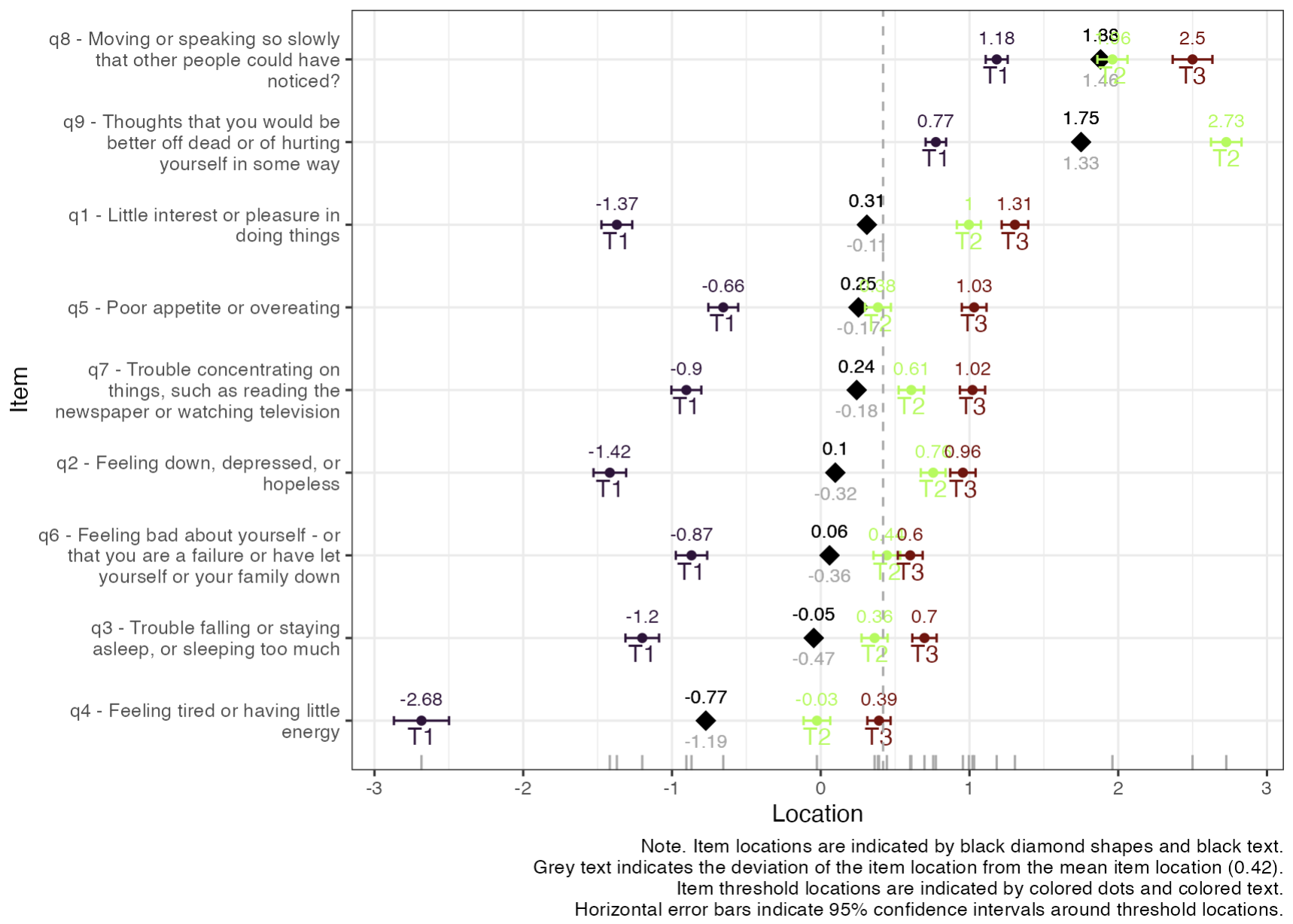
**
